# Excess free volume and structural properties of inert gas condensation synthesized nanoparticles based CuZr nanoglasses

**DOI:** 10.1038/s41598-021-98494-8

**Published:** 2021-09-28

**Authors:** Kaifeng Zheng, Suyue Yuan, Horst Hahn, Paulo S. Branicio

**Affiliations:** 1 Mork Family Department of Chemical Engineering and Materials Science, University of Southern California, 3651 Watt Way, Los Angeles, CA 90089 USA; 2 Institute of Nanotechnology (INT), Karlsruhe Institute of Technology (KIT), 76344 Eggenstein-Leopoldshafen, Germany

**Keywords:** Nanoscale materials, Structural materials, Materials science, Theory and computation, Atomistic models

## Abstract

Nanoglass (NG) as a new structure-tunable material has been investigated using both experiments and computational modeling. Experimentally, inert gas condensation (IGC) is commonly employed to prepare metallic glass (MG) nanoparticles that are consolidated using cold compression to generate an NG. In computational modeling, various methods have been used to generate NGs. However, due to the high computational cost involved, heretofore modeling investigations have not followed the experimental synthesis route. In this work, we use molecular dynamics simulations to generate an NG model by consolidating IGC-prepared Cu_64_Zr_36_ nanoparticles following a workflow similar to that of experiments. The resulting structure is compared with those of NGs produced following two alternative procedures previously used: direct generation employing Voronoi tessellation and consolidation of spherical nanoparticles carved from an MG sample. We focus on the characterization of the excess free volume and the Voronoi polyhedral statistics in order to identify and quantify contrasting features of the glass-glass interfaces in the three NG samples prepared using distinct methods. Results indicate that glass-glass interfaces in IGC-based NGs are thicker and display higher structural contrast with their parent MG structure. Nanoparticle-based methods display excess free volume exceeding 4%, in agreement with experiments. IGC-prepared nanoparticles, which display Cu segregation to their surfaces, generate the highest glass-glass interface excess free volume levels and the largest relative interface volume with excess free volume higher than 3%. Voronoi polyhedral analysis indicates a sharp drop in the full icosahedral motif fraction in the glass-glass interfaces in nanoparticle-based NG as compared to their parent MG.

## Introduction

The multifaceted structure, properties, and the dependence on processing conditions of metallic glasses (MG) have increasingly drawn attention of the materials community since they were reported in the 60’s^1–7^. The synthesis of a bulk MG sample requires the application of extremely high cooling rates on the liquid phase to avoid crystallization and form the amorphous structure. Although the use of low cooling rates has been reported^6,8–10^, the lowest cooling rates to form binary alloys MGs are generally larger than 10^4^ Ks^−1^ ^11^. Due to the unique mechanical and magnetic properties of MG^6,12^, they have been applied in different fields^13–15^. However, their wide spread application is hindered by their common lack of ductility and tendency to fail catastrophically by the generation of critical shear bands resulting in brittle failure^16–18^. Gleiter et al. proposed a nanoglass (NG) concept, an MG with a built-in nanostructure of glass-glass interfaces, which offers an interesting path to tweak MG properties, in particular its mechanical properties^19,20^. The distinctive properties of NGs are rooted at the presence of glass-glass interfaces, which display lower local density and dissimilar short-range order than their MG parent^20–22^. So far, reports on mechanical^21,23^ and electromagnetic properties^21,24–26^ of NGs show they are promising candidates for many engineering applications^27–29^.

The traditional synthesis of NGs, as reported by Gleiter et al.^19,20^ involves two steps: (1) generation of nanoparticles; (2) consolidation of nanoparticles under high pressure (1.5 to 5 GPa) and low, typically room, temperature^26,30^. In the first step, nanoparticles can be conveniently generated by inert gas condensation (IGC)^30–33^. IGC has been applied to a variety of binary systems such as Au–Si, Cu–Sc, Fe–Si, Pd–Si, etc^34^. Chemical segregation to the surfaces of the nanoparticles has been reported to occur during the condensation process^35^.

Atomistic modeling has provided important insights into the structure and the mechanical properties of NG structures^36–44^. In the common procedure followed by modeling simulations, MGs are initially constructed by melting and quenching of an alloy with the same composition as the required NG^39,45^. The generated MG is then used as a reference for construction of the NG structure to be studied. In the preparation of the NG microstructure, different methods can be used. Due to computational efficiency, Voronoi tessellation is a common approach to generate NG structures with mathematically defined planar interfaces^39,46^. NG structures can also be prepared by compression of spherical nanoparticles, which are carved from the MG reference system^42–44,47^. MG nanoparticles generated directly by melting and quenching have also been used to generate NG structures^41,47^. While previous modeling of NGs has provided useful insights, the structure and mechanical behavior of NG samples, produced by consolidation of NG nanoparticles generated by IGC, inspired by the experimental procedure, still needs to be determined.

In this work, we take a first step in the direction of filling the knowledge gap, and characterize the structure of a Cu_64_Zr_36_ NG produced by low temperature high pressure consolidation of amorphous nanoparticles synthesized by IGC, following a procedure recently reported^41^. Detailed analysis of the excess free volume and prevalent polyhedral motifs of the amorphous structure is compared to that of NGs produced by consolidation of MG nanoparticles carved from a bulk MG structure and produced by Voronoi tessellation. Results indicate a significant contrast in the calculated properties among the different samples, in particular in the excess free volume at glass-glass interfaces.

## Methods

MD simulations are performed with the LAMMPS package^48^. An embedded atom model (EAM) fitted to Cu_64_Zr_36_^7^ is used to model atomic interactions. Periodic boundary conditions (PBCs) are imposed along all three Cartesian directions. In-house codes are used in the construction of the face centered cubic (fcc) packing models and the Voronoi tessellated NG. OVITO^49^ and Python scripting are used in the analysis of the simulation results. Initially, a Cu_64_Zr_36_ MG is prepared following the method described in a previous work^39^. A small MG sample containing ~ 10,000 atoms is generated in a simulation box of dimensions  ~ 5.4 × 5.4 × 5.4 nm^3^ under 50 K and 0 GPa. The system is then heated up, melted, and equilibrated at 2000 K for 0.2 ns. Subsequently, it is quenched to 50 K at a rate of 10^10^ K/s. The pressure during the whole process is set at 0 GPa using the NPT ensemble. After the small MG sample is generated, the final MG is generated by replicating the small sample by 4 times in all three dimensions, and annealing at the glass transition temperature *T*_g_,  ~ 800 K for 0.1 ns. The final MG contains  ~ 630,000 atoms. In the preparation of the NG samples, we employ three packing models. In the first model, we use the Voronoi tessellation method to define mathematically the grain microstructure of the NG^39^ with the average grain size set at  ~ 7 nm. The grains are filled up with MG phase extracted randomly from the MG sample. In the second model, an fcc structure is prepared using 32 nanoparticles with  ~ 7 nm diameter, produced by carving a spherically shaped region from the MG sample. In the third model, we use a  ~ 7 nm spherical particle generated by IGC under 4 bars following the procedure described in a recent report^50^. The nanoparticle is replicated and rotated to obtain a set of 32 particles that are used to construct an fcc structure array. After the construction of the models, simulations of cold consolidation are performed under 5 GPa hydrostatic pressure using the NPT ensemble at 50 K. The systems are equilibrated at 5 GPa for 0.1 ns and then relaxed at 0 GPa for another 0.1 ns. Timesteps of 1 fs are used in all simulations.

## Results

### Cold compression consolidation

**Figure 1:**
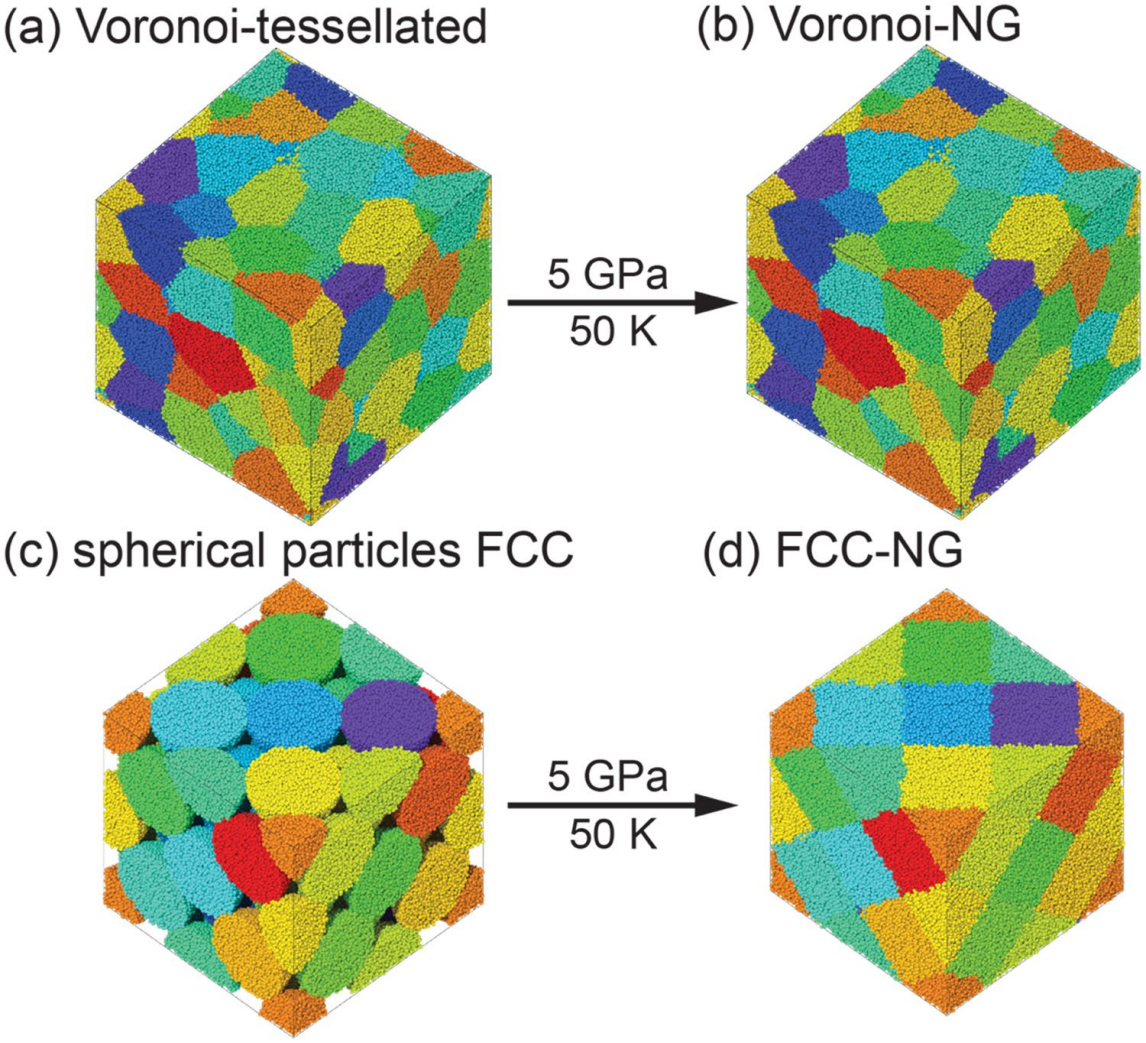
Illustrations of the nanoglass (NG) models generated by cold compression. (**a**)–(**b**) NG produced from Voronoi-tessellated microstructure. (**c**)–(**d**) NG produced from spherical particles arranged in a fcc structure. Colors indicate different nanoparticles. Images created with OVITO version 3.5.3, https://www.ovito.org.

**Figure 2:**
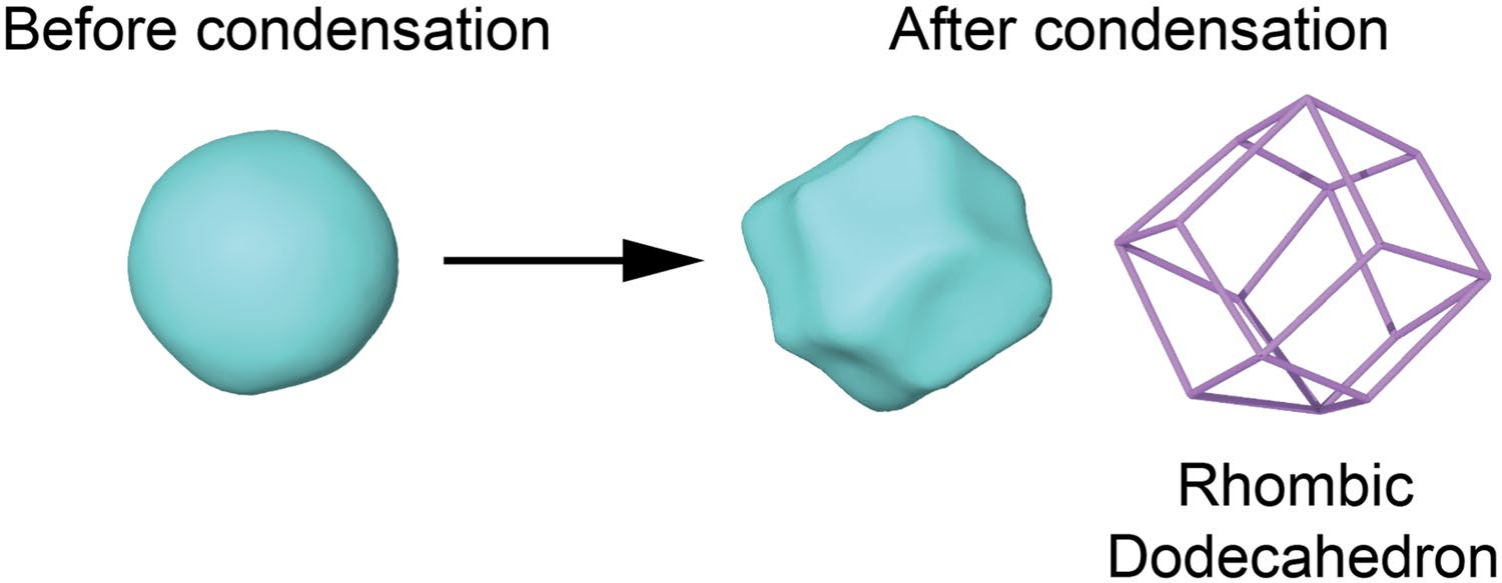
Shape change of spherical nanoparticles in the FCC-NG model during cold compression. After consolidation, the spherical-like nanoparticles adopt a rhombic dodecahedron polyhedron shape. Images created with OVITO version 3.5.3, https://www.ovito.org.

While the cold compression process used to generate the three NG models is similar, the microstructures generated are fundamentally different. Illustrations of the initial and final structures during the cold compression process for the Voronoi-tessellated model (Voronoi-NG) and spherical particle model are shown in Fig. 1. For the spherical particle model, two types of nanoparticles are used to construct the fcc structure as detailed in the previous section. The microstructures of glassy grains and glass-glass interfaces are identical in a macroscopic way for the spherical particle models, as can be seen in Figs. 1c–d. Nonetheless, the nanoparticles in one NG are produced directly by cutting from the bulk MG (MG-NG) while the other is generated by IGC generated nanoparticles (IGC-NG), following closely the experimental route. Thus, topological differences in the amorphous structure are expected to be found in the two nanoparticle-based models. After compression, the voids in the fcc structure vanish and the initially spherical-like particles deform into a rhombic dodecahedron-like shape, with each particle having 12 neighbors, as shown in Fig. 2. One should note that in this study we adopt an fcc packing for the nanoparticles-based NG, which results in the maximum packing factor of 74%. That is identical to what one would obtain from a hexagonal closed packing (hcp). While we follow the experimental route as close as possible, experimentally, such ideal nanoparticles packing may not be achieved, and the nanoparticles may deviate from a well-defined size, resulting in differences compared with the modeling performed here.

**Figure 3:**
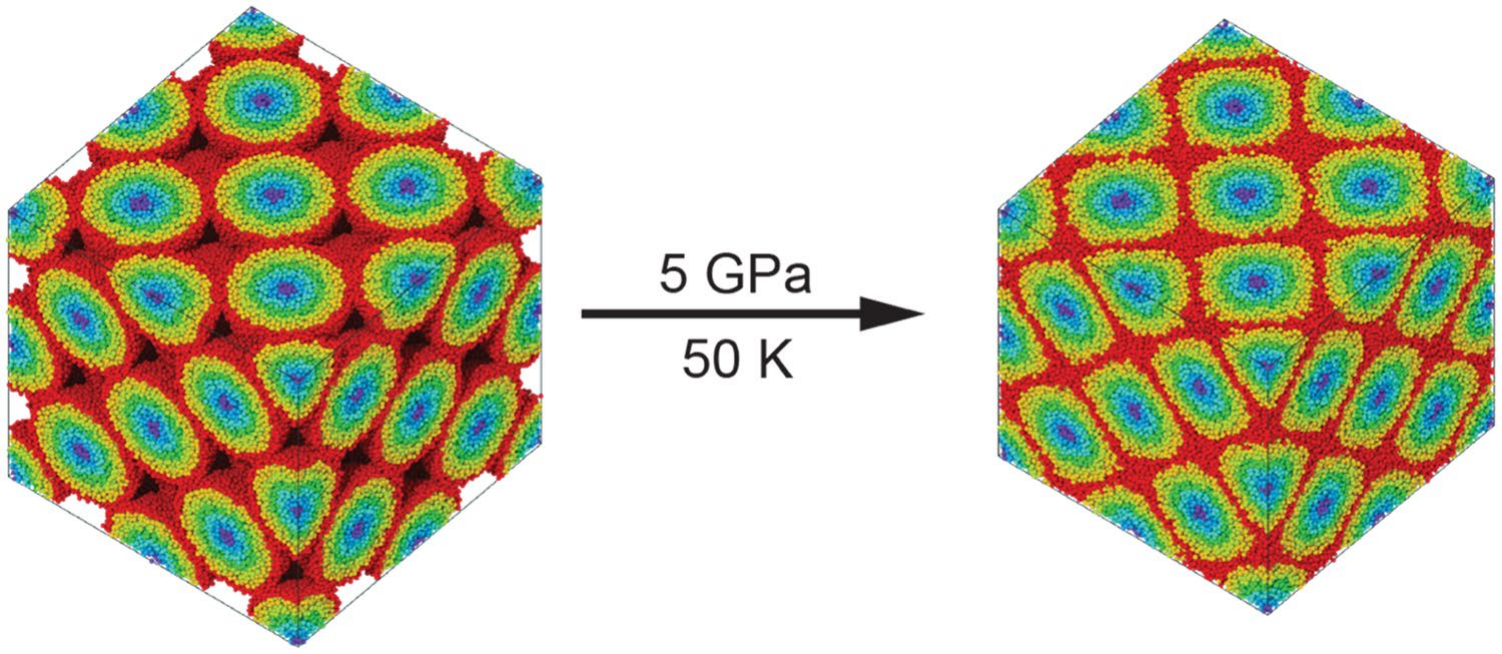
Illustration of the FCC-NG model before and after cold compression with atoms colored according to their initial position in a 7-layered shell structure from the core of each nanoparticle (dark blue) to its surface (red). Images created with OVITO version 3.5.3, https://www.ovito.org.

The glass-glass interfaces in the Voronoi-NG model are flat well defined regions with well-defined thickness of ~ 1.7 nm^39^. Atoms in those regions can thus be conveniently selected based on this well-defined boundary thickness and the local structure analyzed. In contrast, it is challenging to investigate the corresponding properties of glass-glass interfaces in the spherical nanoparticle NG models, since the interfaces have an irregular shape resulting from the deformation of the originally spherical nanoparticles. We then turn to an indirect approach to identify the interfaces and analyze their structure and properties of the spherical nanoparticles model, i.e., tracking the relative position of each interfacial atom with respect to its original position in a superficial layer of the nanoparticle. We split each originally spherical nanoparticle in layers (shells) with a constant thickness from the core position to the surface. This is done as a preprocessing step before the construction of the fcc structure and cold compression consolidation. After defining the center of mass of each particle, each spherical particle is divided into seven spherical layers from its center of mass to the surface with a thickness of 5 Å and the atoms are labeled from layer 1 to layer 7. On account of the surface roughness of the particle, the radius of each particle is approximately 35 Å. The fluctuation of the radius is counted in the center layer (layer 1) and the remaining layers are exactly 5 Å thick. The nanoparticles layers are tracked during the cold compression and illustration of the initial and final structures are shown in Fig. 3, which indicates each one of the 7 layers in a different color following an inverted rainbow gradient. It should be emphasized that while not perfect the layers method is very convenient for spherical like particles based NGs and serves the purpose of this work, which is to compare the interfacial regions of different NGs. For general shaped particles, other accurate methods can be used to identify glass-glass interfaces based on local coordination number methods, such as the quasi-nearest atom^51,52^, order parameters^53,54^, or Voronoi polyhedral based descriptors^39^.

### Excess free volume and structural correlations

**Figure 4:**
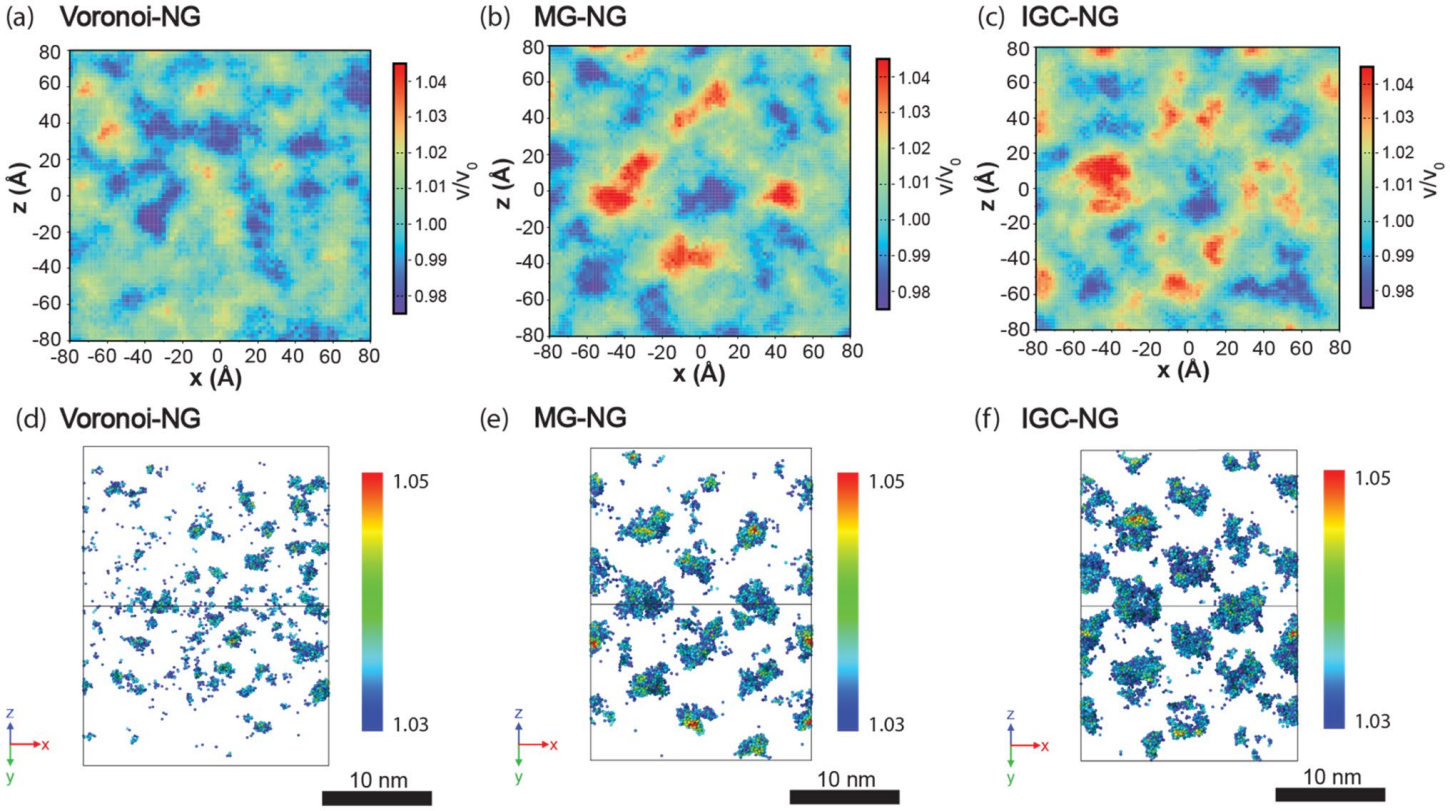
Spatial distribution of excess free volume in the Voronoi-NG model as compared to FCC-NG models based on MG carved particles (MG-NG) and inert gas condensation generated particles (IGC-NG). (**a**)–(**c**) Distribution of atomic volume averaged in a spherical volume of 1 nm radius around each atom, normalized by the bulk MG averaged atomic volume. (**d**)–(**f**) Distribution of atoms with normalized atomic volume greater than 1.03. Images (**d**)–(**f**) created with OVITO version 3.5.3, https://www.ovito.org.

While all NG models possess an amorphous structure, the structure of each one of them is distinct from their MG counterpart because of the introduction of grain boundary-like glass-glass interfaces. Those interfacial regions display a dissimilar amorphous structure and properties when compared to the amorphous structure at inner grains or to that of a bulk MG^21,23,55,56^. In order to understand the microstructure in different NG models, we show the excess free volume distribution profiles of each model using both 2-D and 3-D representations, see Fig. 4. The average local atomic volume in the NGs is calculated at the position of each atom by averaging over neighbor atoms within a spherical volume of 10 Å radius. We map the excess free volume distribution on the NG model MD cells front face, at the *xz* plane and select a 1 nm thick layer along the *y*-direction to average the values. The results shown in Fig. 4a–c, display some regions with large excess free volume, where the average atomic volume is more than 4% from the average value in bulk MG, which is 15.8 Å^3^. The excess free volume distribution of the MG-NG and IGC-NG indicate the existence of both regions with low and high density. The arrangement of those regions with different density displays a clear pattern with high-density regions alternating with low-density regions. The high excess volume regions are contributed by the interfaces (red colored regions). In Fig. 4d–f, we use 3D visualizations to show the distribution of atoms in the regions with excess free volume larger than 3%. A relative volume color-coding from *V*/*V*_0_ = 1.03 to 1.05 is used to show the detailed excess free volume distribution. In the Voronoi-NG, only small sporadic regions are shown. Those excess free volume regions account for 0.6% of the total atoms in the Voronoi-NG model. In contrast, the MG-NG and IGC-NG models display larger regions with higher excess free volume, containing 1.8% and 3.0% of the total number of atoms in their respective systems. For the fcc models, the IGC-NG shows a higher concentration of regions with a high excess free volume than the MG-NG. Arguably, that is rooted at the fact that the particles condensed from the gas phase do not have perfectly spherical shapes and have high surface roughness than the perfectly spherical particles cut from an MG reference system, which have an atomically smooth surface. During the compression, the less ideal spherical nanoparticles generate a rather heterogeneous structure, which is not as tightly packed as the MG-NG. Therefore, the interfaces in the IGC-NG have a lower density than those in the MG-NG.

**Figure 5:**
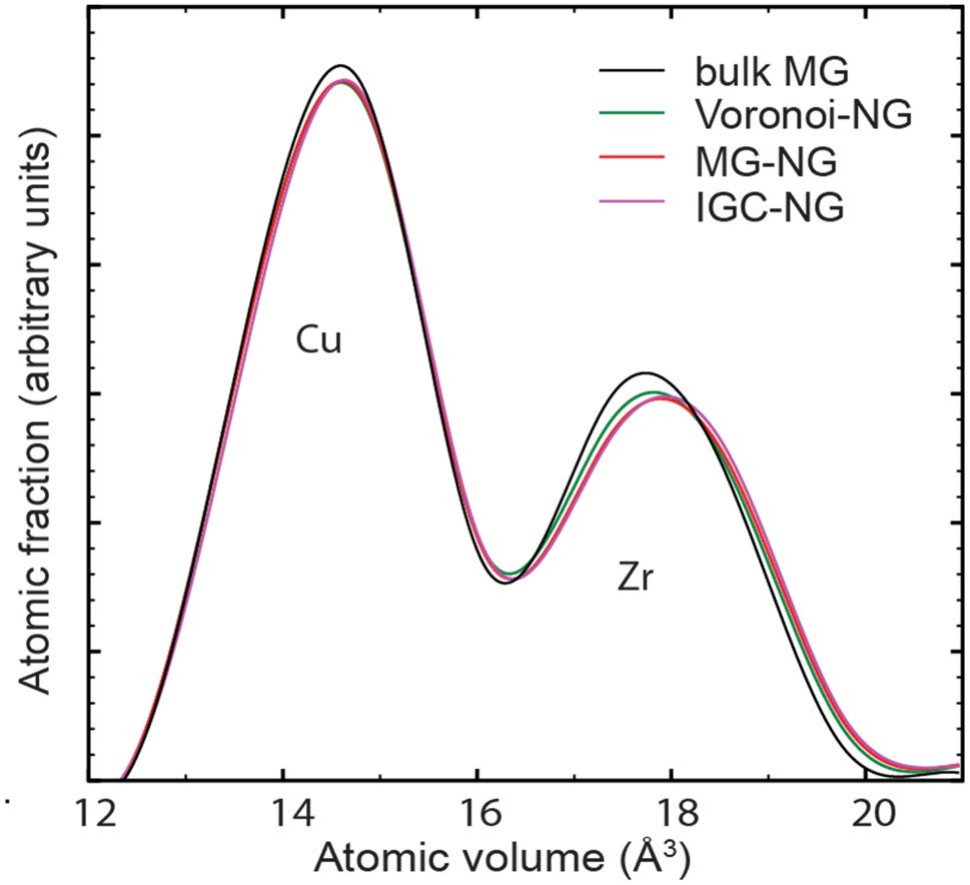
Voronoi atomic volume distribution in different NG models as compared to their bulk MG parent. The peaks corresponding to Cu and Zr contributions are indicated.

To better understand the excess free volume distribution in the NGs, we calculate distribution of Voronoi atomic volumes in all NG models and compare to that in the reference bulk MG. The curves are shown in Fig. 5 and display the distribution curves of atomic volume for the Voronoi-NG, MG-NG, IGC-NG, and bulk MG. Among all amorphous samples Cu atoms have an average atomic volume of ~ 14.7 Å^3^ while Zr atoms have a much larger average volume of ~ 17.8 Å^3^. On observation one can note that the distribution curves for the bulk MG display a visible deviation from the three NG systems. In particular, NGs have a relatively larger average Zr atomic volume than that in the bulk MG, ~ 17.9 Å^3^ vs. ~ 17.75 Å^3^. However, the distribution of atomic volumes for Cu atoms indicates a barely visible shift to higher atomic volumes of the NG as compared to the bulk MG reference.

**Figure 6:**
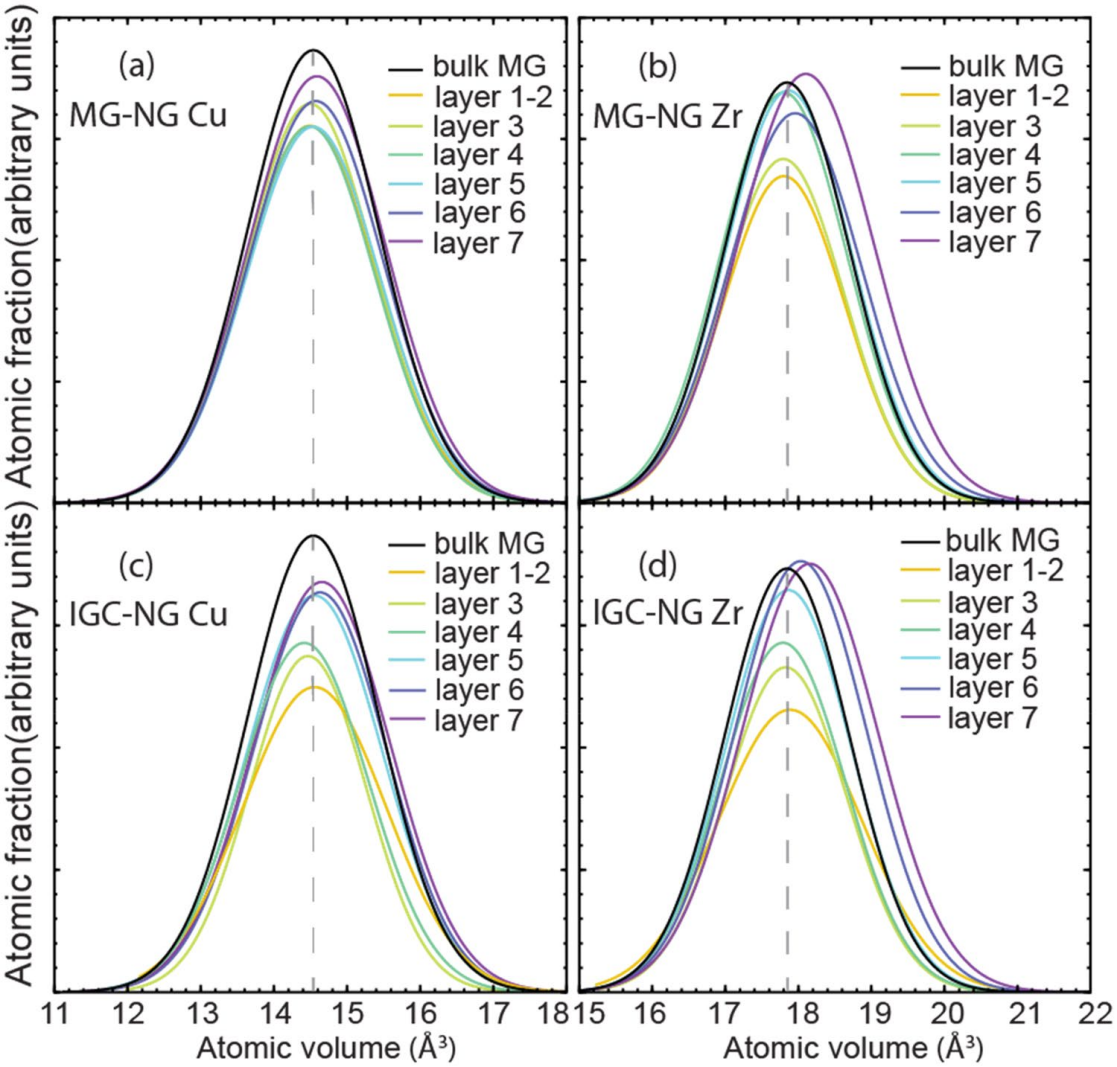
Voronoi atomic volume distribution in each layer of (**a**)–(**b**) IGC-NG and (**c**)–(**d**) MG-NG. The dash line indicates the peak position of the bulk MG curve. The atomic fraction is calculated by dividing the number of atoms of specific element in the selected layer. Each curve is fitted using a Gaussian distribution function.

To develop a better understanding of the spatial variation in the statistics of atomic volume in the MG-NG and IGC-NG models, we calculated their statistics in each one of the different layers. The resulting curves are shown in Fig. 6 and show separately the distributions for Cu and Zr atoms. One can note that the peaks of distributions for layers 1 to 5 in both MG-NG and IGC-NG models display slight to no deviation from that in the reference bulk MG, indicated by the dashed vertical line. However, the curve peaks for layers 6 and 7 for both MG-NG and IGC-NG models indicate a prominent deviation from the curve of bulk MG, especially for the atomic volume of Zr. For the IGC-NG, Cu and Zr curves both show a positive deviation from the bulk MG for layers 6 and 7. That indicates the source of the excess free volume at interfaces, which are distributed along the layers 6 and 7, i.e., within a 1 nm thick layer from the original surface of the nanoparticles. As shown in Fig. 3 after consolidation the layers 6 and 7 form an irregular shaped volume that connects the glassy grain cores, which contain a relatively unaffected amorphous structure.

**Figure 7:**
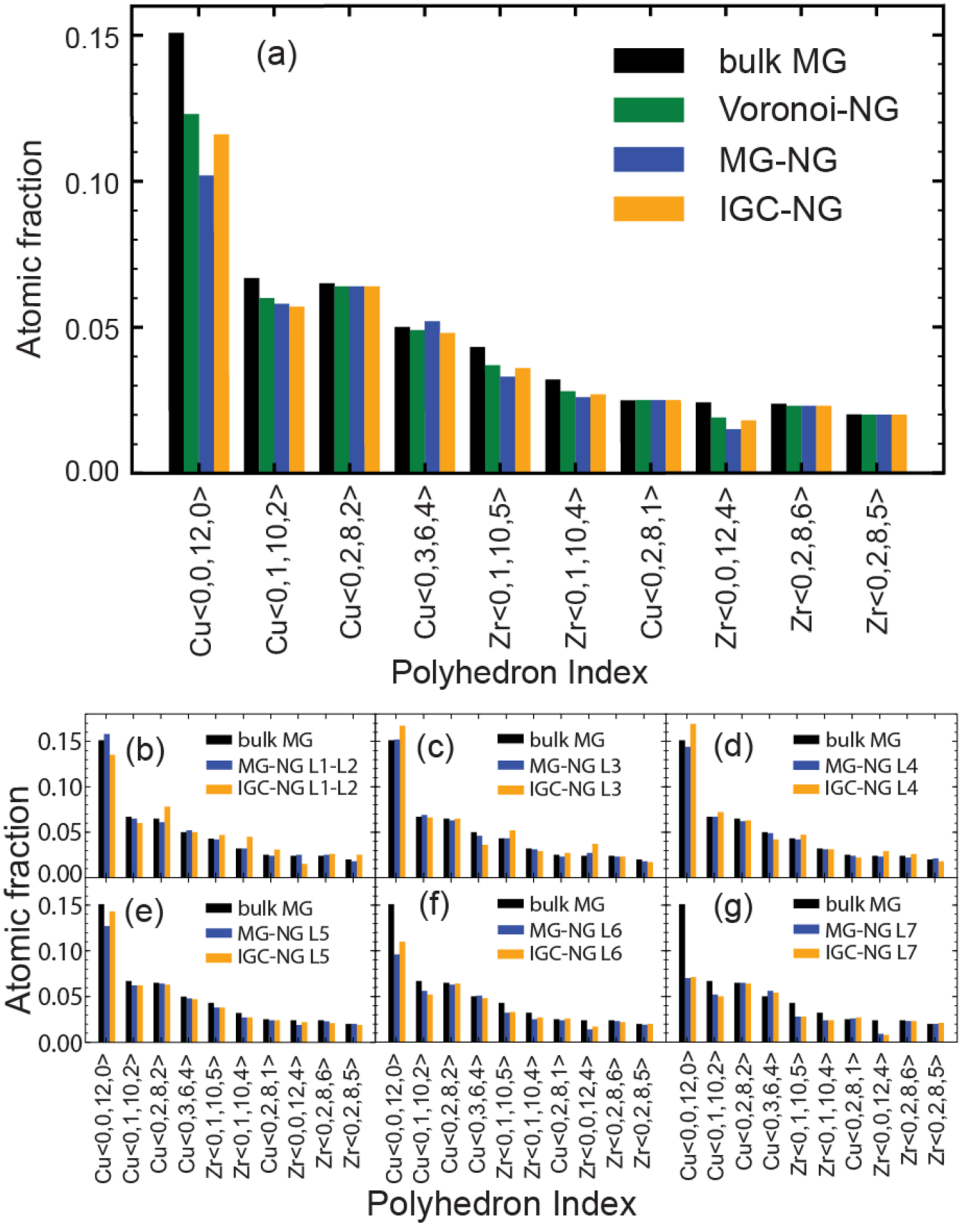
Statistics of the most prominent Voronoi polyhedrons in the Voronoi-NG, MG-NG, IGC-NG, and bulk MG models. (**a**) Statistics of the most prominent Voronoi polyhedrons in different NG and MG models (**b**)–(**g**) Corresponding statistics of the MG-NG and IGC-NG models split in seven layers from the core of the particles to their surface.

Besides excess free volume the glass-glass interfaces introduce changes to the medium range order of the amorphous structure. That is often quantified by calculating the statistics of the most prominent atomic Vonoroi polyhedra. In Fig. 7 we show the statistics of the most prominent polyhedra in the MG and NG samples. The polyhedron indexes are denoted as α < *i,j,k,l* > , where α represents the center atom of the polyhedron cage, and *i, j, k*, and *l* are the number of polygon faces with 3, 4, 5 and 6 edges. Cu rich Cu-Zr MG most prominent polyhedron is Cu full icosahedron (Cu FI), Cu < 0,0,12,0 > ^39,44,47^. The results show that NGs have a lower atomic fraction of Cu FI compared to the bulk MG, as shown in Fig. 7a. The atomic fractions of polyhedrons analyzed separately in each layer for the MG-NG and IGC-NG are shown in Fig. 7b–g. Each plot is compared with the reference atomic fraction of polyhedra of the MG reference structure. For layers 6 and 7, shown in Fig. 7f,g, the atomic fraction of Cu FI declines abruptly. This decline is associated with the changes in the amorphous structure at the glass-glass interfaces^39,44,47^. That demonstrates that the layers 6 and 7 are within the interfacial region. One should also note that layer 5 indicates a small drop in Cu FI, which suggests that the interfaces in NG constructed from nanoparticles may originate from layer 5, 1.5 nm from the original nanoparticle surface.

## Discussions

### Structural features of different NG models

The results presented show noticeable differences between the microstructures of the Voronoi-NG model and the MG-NG and IGC-NG nanoparticle-based models. According to the spatial distribution of the excess free volume shown in Fig. 4, the Voronoi-NG displays dispersed and smaller regions with high excess free volume than the other two cases. That is a consequence of the stochastic distribution and geometry of the glass-glass interfaces in the Voronoi-NG model, which are flat and have a fixed thickness, in contrast with that of the particle-based NG models, as indicated in Figs. 1(b) and 3. In the Voronoi-NG, regions with high excess free volume are located at triple junctions of the microstructure and have limited volumes that are independent of the particle size. In contrast, in the particle-based NG models considered here, high excess free volumes are found in the interstitial regions of the FCC superstructure and depend directly on particle size. This explains the profiles shown in Fig. 4 and also indicates the source of the differences in the curves of overall distribution of Voronoi atomic volumes, shown in Fig. 5. The curves for MG-NG and IGC-NG shown in Fig. 5 display a shift to higher Zr atomic volumes as compared to the Voronoi-NG. Additionally, the results shown in Fig. 4 show that between the nanoparticle-based models, a larger region with higher excess free volume is identified in the IGC-NG model (3% of atoms) than in the MG-NG model (1.8% of atoms). This result suggests that small deviations in the spherical shape of the nanoparticles, the inherent surface roughness, and the presence of Cu surface segregation in the IGC generated nanoparticles lead to enhanced excess free volume at glass-glass interfaces. Though one can hardly recognize the distinction between the two nanoparticle-based models based on the overall atomic volume distribution shown in Fig. 5, the contrast is clear in the data shown in Fig. 6 for the different particle layers. In comparison, the IGC-NG model shows a rather distinct variance in curves between core (layers 1–2) and interface (layers 6–7) regions than the MG-NG model. According to the Voronoi polyhedra statistics shown in Fig. 7a, no sharp difference is found among the statistics of the three models, which implies that they have similar overall short-to-medium range order. The results shown in Fig. 7 are consistent with those shown in Fig. 5 indicating similar overall structure and atomic Voronoi volumes. However, the results shown in Figs. 3, 4 and 6 indicate that the three models have contrasting glass-glass interfaces shapes and volumes and contrasting excess free volumes.

NG models previously proposed and investigated can be mostly classified as either Voronoi-NG or MG-NG like models^17,36–44,47^. Voronoi-NG like models have been proposed and applied in several investigations of the structure and mechanical behavior of NGs^17,36–40^. In contrast, a few investigations on MG-NG like models are mostly focused on the properties of the glass-glass interfaces^41–44,47^. In agreement with the results presented here, studies of flat glass-glass interfaces in Voronoi-NG models indicate about 1 – 2% excess free volumes^37,39^. It should be highlighted that experiments indicate excess free volumes as high as 6%^25,29^. Interestingly, a previous work pointed out that the amount of excess free volume located at flat glass-glass interfaces in an NG was correlated to the observed flow strain of the corresponding homogeneous MG subjected to tensile loading^57^, i.e., Ge MG deformed at 300 K displayed a flow strain of 8% while the interfaces in Ge NG showed a 8% excess free volume. However, the results of this work indicate that the excess free volume in non-flat glass-glass-interfaces of particle based NGs can display excess free volumes well above above that predicted by using flat interfaces^37,39^. The results also suggest a heterogeneous distribution of excess free volume throughout the non-flat interfaces with higher excess free volumes at three and four particle joint regions. That implies an expected dependence on grain size of the excess free volume distribution in NGs produced by cold compression of glassy particles.

The statistics of Voronoi polyhedra is a fingerprint of the short-range order in an MG. The most prominent polyhedron in Cu rich CuZr MG is the Cu FI. A low fraction of Cu FI was reported in the glass-glass interfaces of NGs in both Voronoi-NG^36,39^ and MG-NG^42,44^ models in agreement with the results shown in Fig. 7. It should be mentioned that the effect of Cu surface segregation was considered explicitly in several previous investigations of NG models^36,41,47,55^. However, to the best of our knowledge there is no report on other effects of IGC process such as variations in the particle spherical shape and surface roughness. As the results indicate those subtle effects result in significant contrast between the glass-glass interfaces in MG-NG and IGC-NG as shown in Figs. 4 and 6 that were previously underestimated.

### Experimental synthesis of NGs and comparison with modeling

It is instructive to compare the NG models investigated in this computational work with those synthesized in experiments. Various experimental methods have been employed in the generation of NG samples. As mentioned previously, one common way to generate an NG is by consolidation of glassy nanoparticles produced by IGC. Depending on the NG alloy system, one may combine IGC with different vapor producing techniques such as thermo evaporation^58^, magnetron sputtering^24,59^, and pulsed laser ablation^60^. The generated nanoparticles have spherical-like shapes and average sizes typically in the range of 4 to 10 nm^22,25,59^ even though average nanoparticles as high as 40 nm have been reported^60^. These nanoparticles are then consolidated by cold compression to form an NG^19,56,61^. The IGC-NG model investigated in this work is constructed following closely the experimental route and consequently is expected to display microstructures representative to the experimental NG samples. Gleiter et al.^25,29^ found that in the Sc-Fe NG samples, the free volume in the glass-glass interfaces is about 6% higher than the free volume in the adjacent glassy regions. Comparing this value with the excess free volumes predicted in the three NG models, we may conclude that the IGC-NG model can best capture the microstructural features of experimental NG samples and match the experimentally reported high values of the excess free volume at glass-glass interfaces. Although in this simulation work, we did not consider the possibility of oxidation of the samples, it may be an important factor in experiments. When MG particles contain oxidizable elements, oxidation may occur and have an effect on the properties of the material. Oxidation will result in glassy oxide interfaces that may significantly affect the chemical composition and microstructure of the resultant NGs^62,63^. It is worth mentioning that the grain packing in experimental NGs usually depends on the specific consolidation path and could differ from the perfect fcc packing pattern in our models and the resulting distribution pattern of excess free volume.

Besides the common IGC followed by high pressure consolidation, an NG sample can also be prepared directly by using physical vapor deposition methods such as magnetron sputtering^31,64^. In this case, the resulting NG grown thin film produced has a columnar structure. NG produced directly by magnetron sputtering are synthesized under a much lower pressure than that in the IGC process, which might result in a different chemical segregation state at glass-glass interfaces. NG samples synthesized in such a way display an architecture rather similar to that of Voronoi-NG thin films^36–39,65^. Electrodeposition is a third approach to prepare NGs^32,66^. These NGs have a hierarchical structure with nano-sized particles combined into micro-sized clusters, forming island-like grains. To the best of the authors knowledge, there is currently no NG simulation models that mimics such hierarchical structure.

### Excess free volume, atomic diffusion and the contrasting changes in atomic volumes for Cu and Zr species

The origins of the excess free volume and the discrepancies in the atomic volume for different species shown in Figs. 5 and 6 deserve to be further discussed. As shown in Fig. 5, MG-NG and IGC-NG have noticeable larger average Zr atomic volume than that in the bulk MG, while only a barely noticeable shift is found in the Cu atomic volume distribution curve. When combining this observation with the results in Fig. 6, we can conclude that the increase in the excess free volume of the NG systems is linked to the increase in Zr atomic volume at interface regions, i.e., layers 6 and 7. This can be traced to the higher diffusion coefficients of Cu as compared to that of Zr in Zr-based amorphous alloys^67,68^. During the consolidation of the MG-NG and IGC-NG samples, Cu atoms tend to diffuse to the surface while the sample absorbs vacancies and introduce excess free volume into the material by continuous vacancy absorption and vacancy diffusion. That has three consequences, Cu surface segregation, increase in the overall excess free volume of the sample, and contrast between the atomic volumes of Cu and Zr atoms. Consequently, there is no evident difference in the average atomic volume of Cu between different layers. In contrast, the average atomic volume of Zr atoms presents a more distinct spatial variation, contributing to most of the excess free volume in the NG interfacial regions, as shown in Fig. 6.

## Conclusion

In this work, we evaluated the excess free volumes and structural correlations in three NG models constructed using distinct methods, i.e., Voronoi-NG, MG-NG, and IGC-NG, by using MD simulations. Results show that the distribution of excess free volume in all models follow a well-defined pattern with regions with low relative density (high excess free volumes) associated with glass-glass interface regions. However, in contrast with the Voronoi-NG, particle-based MG-NG and IGC-NG models display large regions with excess free volumes higher than 3% due to the assimilation of interstitial voids and absorption of vacancies during the consolidation process. Notably, a comparison of nanoparticle-based NGs indicates that the IGC-NG exhibits larger volumes with excess free volume than the MG-NG, arguably because of the less ideal spherical particle shapes, higher surface roughness, and Cu surface segregation on the nanoparticles generated by IGC. This work critically assesses the excess free volume distribution and the structure of different NG models in the light of experimental reports and shed light on the atomistic mechanisms giving rise to the appearance of regions with abnormal excess free volumes of 4% or more. That points out that in order to generate realistic NG models with excess free volumes in excellent agreement with experiments, it is imperative to follow the experimental workflow in the production of NGs. In future simulation work of NGs, one may consider using the IGC-NG model to obtain results that closely follow those of NGs generated by IGC in experiments.

## Disclaimer

This report was prepared as an account of work sponsored by an agency of the United States Government. Neither the United States Government nor any agency thereof, nor any of their employees, makes any warranty, express or implied, or assumes any legal liability or responsibility for the accuracy, completeness, or usefulness of any information, apparatus, product, or process dis- closed, or represents that its use would not infringe privately owned rights. Reference herein to any specific commercial product, process, or service by trade name, trademark, manufacturer, or otherwise does not necessarily constitute or imply its endorsement, recommendation, or favoring by the United States Government or any agency thereof. The views and opinions of authors expressed herein do not necessarily state or reflect those of the United States Government or any agency.
